# Comparison of gut bacterial communities of *Hyphantriacunea* Drury (Lepidoptera, Arctiidae), based on 16S rRNA full-length sequencing

**DOI:** 10.3897/BDJ.11.e98143

**Published:** 2023-05-10

**Authors:** Hui Gao, Sai Jiang, Yinan Wang, Meng Hu, Yuyan Xue, Bing Cao, Hailong Dou, Ran Li, Xianfeng Yi, Lina Jiang, Bin Zhang, Yujian Li

**Affiliations:** 1 School of Life Sciences, Qufu Normal University, Qufu, China School of Life Sciences, Qufu Normal University Qufu China; 2 School of Life Sciences, Shandong University, Qingdao, China School of Life Sciences, Shandong University Qingdao China; 3 Forestry Protection and Development Service Center of Jining City, Jining, China Forestry Protection and Development Service Center of Jining City Jining China; 4 Qufu Bureau of Natural Resources and Planning, Qufu, China Qufu Bureau of Natural Resources and Planning Qufu China; 5 Animal Husbandry and Fisheries Development Centre of Tengzhou, Tengzhou, China Animal Husbandry and Fisheries Development Centre of Tengzhou Tengzhou China; 6 College of Life Sciences and Technology, Inner Mongolia Normal University, Hohhot, Inner Mongolia Autonomous Region, China College of Life Sciences and Technology, Inner Mongolia Normal University Hohhot, Inner Mongolia Autonomous Region China

**Keywords:** *Hyphantriacunea*, intestinal microbiome, 16S rRNA full-length gene, host diet, full-length sequencing

## Abstract

There are a large number of microorganisms in the gut of insects, which form a symbiotic relationship with the host during the long-term co-evolution process and have a significant impact on the host's nutrition, physiology, development, immunity, stress tolerance and other aspects. However, the composition of the gut microbes of *Hyphantriacunea* remains unclear. In order to investigate the difference and diversity of intestinal microbiota of *H.cunea* larvae feeding on different host plants, we used PacBio sequencing technology for the first time to sequence the 16S rRNA full-length gene of the intestinal microbiota of *H.cunea*. The species classification, β diversity and function of intestinal microflora of the 5^th^ instar larvae of four species of *H.cunea* feeding on apricot, plum, redbud and Chinese ash were analysed. The results showed that a total of nine phyla and 65 genera were identified by PacBio sequencing, amongst which Firmicutes was the dominant phylum and *Enterococcus* was the dominant genus, with an average relative abundance of 59.29% and 52.16%, respectively. PERMANOVA analysis and cluster heat map showed that the intestinal microbiomes of *H.cunea* larvae, fed on different hosts, were significantly different. LEfSe analysis confirmed the effect of host diet on intestinal community structure and PICRUSt2 analysis showed that most of the predictive functions were closely related to material transport and synthetic, metabolic and cellular processes. The results of this study laid a foundation for revealing the interaction between the intestinal microorganisms of *H.cunea* and its hosts and provided ideas for exploring new green prevention and control strategies of *H.cunea*.

## Introduction

The gut of an insect is home to a vast and diverse microbial community. In the process of long-term co-evolution with the host, intestinal microbes have also formed extremely diverse population structures and biological functions, playing an important role for insect survival (Ayyasamy et al. 2021), development (Elgart et al. 2016), reproduction, mating (Wei et al. 2021), immunity (Yao et al. 2018), metabolism (Ceja-Navarro et al. 2015), cold tolerance (Mueller et al. 2011), heat resistance (Dunbar et al. 2007), degradation of exogenous toxins (Chen et al. 2020, Jiang et al. 2021), antioxidant (Jiang et al. 2021), adaptation to the environment (Elgart et al. 2016, Ayyasamy et al. 2021), hormone signal transduction (Zeng et al. 2020) and absorption of nutrients (Wang et al. 2016) etc.

*Hyphantriacunea* Drury (Lepidoptera, Arctiidae) is a plant quarantine pest, native to North America (Bai et al. 2020). It has caused great harm to agriculture and forestry because of its strong adaptability, fast propagation speed and wide host range. *H.cunea* mainly feeds on plant leaves with larvae and causes harm. In serious cases, it can eat all the leaves of the host plant and eat the bark, thus weakening the ability of trees to resist damage and stress and seriously affecting the survival and growth of trees. *H.cunea* has a wide and varied diet; studies have shown that the selection of the insect is regulated by the chemosensory system (Obiero et al. 2014) and a large expansion of the chemosensory gene family of *H.cunea* makes it have multiple feeding habits (Chen et al. 2020). At the same time, the enhanced utilisation capacity of new nutrient sources also promotes the rapid adaptation and diffusion of this species in its whole range (Wu et al. 2019), thus causing great damage to agriculture, forestry and ecological environment.

At present, there are many prevention and control measures for *H.cunea*, such as quarantine, webs clearance, light trapping, chemical control, microbial insecticide (Wang et al. 2021), multi-species trapping (Brockerhoff et al. 2013), research and development of transgenic insect-resistant plants (Yang et al. 2016), RNA interference (Wang et al. 2019) etc. However, large-scale spraying of pesticides will kill the natural enemies of the *H.cunea*, increasing the resistance of *H.cunea* itself and destroying the ecological environment. As the natural enemy of *H.cunea*, *Chouioiacunea* Yang has the advantages of environmental friendliness, simple and convenient artificial mass breeding technology, easy to operate etc. Dong conducted bee release experiments in Neihuang County and achieved good results (Dong 2021). However, biological control also has disadvantages, such as higher cost and the control effect is susceptible to environmental conditions (Zheng et al. 2012). Some scholars predicted that the potential distribution of *H.cunea* in the Northern Hemisphere (North America, Europe and Asia) is expected to increase gradually over time (Ge et al. 2019). The prevention and control situation of *H.cunea* is still serious, so it is urgent to explore new control methods of *H.cunea*.

With the development of high-throughput sequencing technology and omics technology, a growing number of researchers are studying insect gut microbes (Cao and Ning 2018). For example, *Lactobacillusplantarum* can not only affect the mating preference of *Drosophilamelanogaster*, but also affect the mutual recognition before mating by prolonging the mating latency (Wei et al. 2021). The gut microbiota of two Lepidopteran herbivores *Dendrolimussuperans* and *Lymantriadispar* may become altered under plant defensive compound‐induced stresses (Zeng et al. 2020). The antioxidant and detoxification defence systems, as well as the gut microbiota of *H.cunea* larvae, constitute an effective counter-defence regulatory mechanism against the secondary metabolites, based phytochemical defence (Jiang et al. 2021). In the presence of exogenous toxins, cadmium and selenium, honeybee gut microbes can not only remove toxins from the gut, but also respond to challenges by upregulation of metabolites related to detoxification (Rothman et al. 2019).

There ismore and more research on the gut microbiota of insects, but the existing research on the intestinal microbiota of *H.cunea* only stays in the next-generation sequencing stage. Compared to the V3–V4 region of the 16S rRNA gene amplicon, full-length sequencing has higher resolution and fewer inaccurate sequences (Burke and Darling 2016). At the same time, this technology has an advantage of longer reads which can easily sequence complete 16S rRNA gene or other marker genes. This advantage can help scientists carry out a phylogenetic microbial community profiling with higher resolution (Yang et al. 2021). Studies have shown that full-length sequencing can be used to detect rarer species (Deutscher et al. 2018) and the accuracy of full-length sequencing annotation was significantly improved compared with that of the short V3-V4 region sequencing (Wang et al. 2021). The V4 region of 16S rRNA gene sequencing has the advantage of detecting archaea. However, this group often occupies low abundance and has seldom been suggested to play important roles in insect hosts (Yang et al. 2021). With the declining price of third-generation sequencing, we prefer to recommend using this technology to sequence full-length of 16S rRNA gene in insect microbiome research. In this study, the full-length 16S rRNA of intestinal microbiota was detected by PacBio third-generation sequencing technology in order to clarify the diversity and unity of intestinal microbiota in larvae of *H.cunea* with different diets, thus providing new ideas for the control of *H.cunea*.

## Methods

### Insect rearing and sample processing

In this study, larvae of *H.cunea* were selected as experimental materials, which fed on four host plants: apricot (A), plum (P), redbud (R) and Chinese ash (CA). The experimental materials were collected in September 2021 on the campus of Qufu Normal University and reared with fresh leaves in an indoor artificial climate chamber at 70 ± 5% RH and 25 ± 1°C under a 14:10 L: D photoperiod (Jiang et al. 2021). The 5^th^ instar larvae were selected as experimental insects.

The 5^th^ instar larvae of *H.cunea* were dissected on a super clean workbench. After sterilisation with an autoclave steriliser, the anatomical tools were irradiated with an ultraviolet lamp for 30min. The body surface of the larva was disinfected with 75% alcohol for 90 s and then cleaned with sterile water for 3 times. The complete intestine was removed and placed into a sterilised 2 ml centrifuge tube and stored at -80°C. Every 10 larva intestines were taken as one sample, each treatment had three replicates.

### DNA extraction

The DNA was extracted with the TGuide S96 Magnetic Soil /Stool DNA Kit (Tiangen Biotech (Beijing) Co., Ltd.) according to the manufacturer's instructions. The DNA concentration of the samples was measured with the Qubit dsDNA HS Assay Kit and Qubit 4.0 Fluorometer (Invitrogen, Thermo Fisher Scientific, Oregon, USA).

### PCR amplification and PacBio sequencing of bacterial 16S rRNA gene

The 27F: AGRGTTTGATYNTGGCTCAG and 1492R: TASGGHTACCTTGTTASGACTT universal primer set was used to amplify the full-length 16S rRNA gene from the genomic DNA, extracted from each sample. Both the forward and reverse 16S rRNA gene primers, were tailed with sample-specific PacBio barcode sequences to allow for multiplexed sequencing. We chose to use barcoded primers because this reduces chimera formation as compared to the alternative protocol in which primers are added in a second PCR reaction. The KOO One PCR Master Mix (TOYOBOLife Science) was used to perform 25 cycles of PCR amplification, with initial denaturation at 95°C for 2 min, followed by 25 cycles of denaturation at 98°C for 10 s, annealing at 55°C for 30 s and extension at 72°C for 1 min 30 s, with a final step at 72°C for 2 min. The total of PCR amplicons were purified with Agencourt AMPure XP Beads (Beckman Coulter, Indianapolis, IN) and quantified using the Qubit dsDNA HS Assay Kit and Qubit 4.0 Fluorometer (Invitrogen, Thermo Fisher Scientific, Oregon, USA). After the individual quantification step, amplicons were pooled in equal amounts. SMRTbell libraries were prepared from the amplified DNA by SMRTbell Express Template Prep Kit 2.0 according to the manufacturer's instructions (Pacific Biosciences). Purified SMRTbell libraries from the pooled and barcoded samples were sequenced on a single PacBio Sequel II 8M cell using the Sequel II Sequencing kit 2.0. Shenzhen Wekemo Technology Group Co., Ltd. was entrusted to build the database and we used PacBio platform for sequencing (Caporaso 2010, Bokulich 2013, Jovel 2016).

### Statistical and bioinformatics analysis

After the base-calling analysis, the original data files were transformed into FASTQ format. QIIME2 (Quantitative Insights Into Microbial Ecology) package was used for quality control, denoising, stitching and chimerism removal of all original sequences of all samples to cluster tags at a similarity level of 99% and to obtain OTU (operational taxonomic units) (Edgar 2013). Sequences identified as chloroplast, mitochondrial sequences and sequences that did not align to bacteria were removed from the dataset. The taxonomic annotation of OTU was performed, based on the Silva (https://www.arb-silva.de) database. We used Analysis of Variance (ANOVA) to assess differences in community richness amongst different feeding groups. We used PCoA analysis and NMDS analysis, based on Bray–Curtis dissimilarity to analyse the bacterial community data. Stress values were used to assess the quality of NMDS representation; stress values < 0.2 are considered a good representation of the data, while those > 0.3 are considered invalid (Quinn and Keough 2002). Linear Discriminant Analysis (LDA) was used to screen the biomarkers for statistical differences between different groups with LDA scores greater than 4. We used PICRUSt to predict the composition of known gut microbial gene functions (Langille et al. 2013).

## Results

### Analysis of 16S rRNA sequencing results

**Figure 1.**
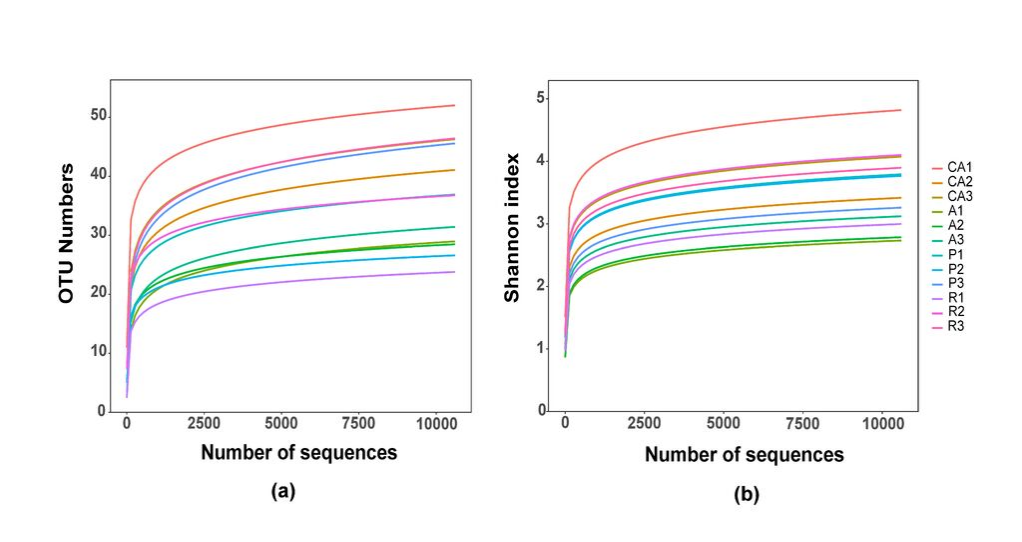
The rarefaction curves show that the sequencing volume is sufficient. (**a**) Sample rarefaction curves; (**b**) Shannon Index rarefaction curves. CA, Chinese ash-feeding; A, apricot-feeding; P, plum-feeding; R, redbud-feeding.

A total of 301,266 original reads were obtained from 12 samples of *H.cunea* larvae with redundancy removed. The lowest number of sample sequence was 11,019. After quality control, 271,869 reads were retained from the original 301,266 reads (Table 1). Cluster analysis revealed 362 OTUs, including nine phyla, 69 genera and 103 species. With the increase of sequence depth, both Shannon Index rarefaction curves (Fig. 1a) and sample rarefaction curves (Fig. 1b) did not increase significantly, indicating that the sequencing volume was sufficient.

### Comparison of intestinal microbial diversity of Hyphantriacunea fed on different host plants

**Figure 2.**
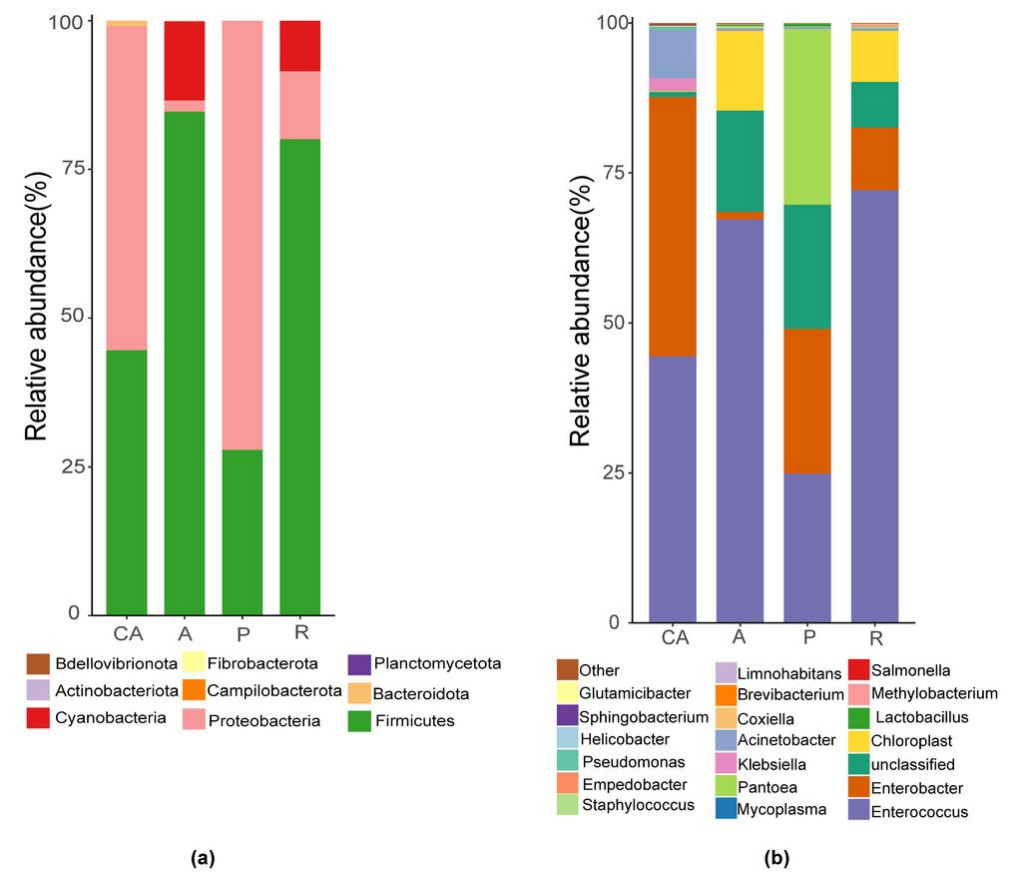
Histogram of intestinal microorganisms of *Hyphantriacunea* larvae at the level of phylum (**a**) and the level of the top 20 genera with the highest abundance (**b**). Different colours represent different species, and the height of the colour block indicates the proportion of the species in relative abundance. Other species are combined as "Other" and unannotated classification dates are incorporated as "Unknown".

The OTUs obtained were compared with the microbial grouping database to obtain the species classification information corresponding to each OTU. The microbial composition of each sample was recorded at levels of phylum, class, order, family, genus and species. We found nine abundant phyla (Fig. 2a), including Firmicutes, Proteobacteria, Cyanobacteria, Fibrobacterota etc. Firmicutes and Proteobacteria were the dominant phyla. Firmicutes was the dominant phylum in the apricot feeding group (84.7%) and redbud feeding group (80.1%), Proteobacteria was the dominant phylum in the Chinese ash feeding group (54.5%) and plum feeding group (72.1%). The relative abundance of Cyanobacteria in the apricot feeding group was the highest (14.26%). In addition, some special phyla existed only in individual feeding groups, for example, a small number of Bdellovibrionota was annotated in the plum feeding group. At the genus level, a total of 65 genera were annotated and the top 20 genera with the highest abundance were selected to draw a histogram (Fig. 2b). Amongst them, *Enterococcus* was the absolute dominant genus. The relative abundance of *Enterococcus* annotated in the redbud feeding group was the highest (72.1%), followed by the apricot feeding group (67.2%) and the relative abundance of plum feeding group was the lowest (24.9%). *Enterobacter* was annotated in all groups, with the highest relative abundance in Chinese ash feeding group (43.26%). *Acinetobacter*, *Pseudomonas* and *Staphylococcus* were annotated in each feeding group, the most *Acinetobacter* and *Pseudomonas* were annotated in Chinese ash feeding group (8.06% and 0.51%, respectively) and the most *Staphylococcus* was annotated in apricot feeding group (0.38%).

**Figure 3.**
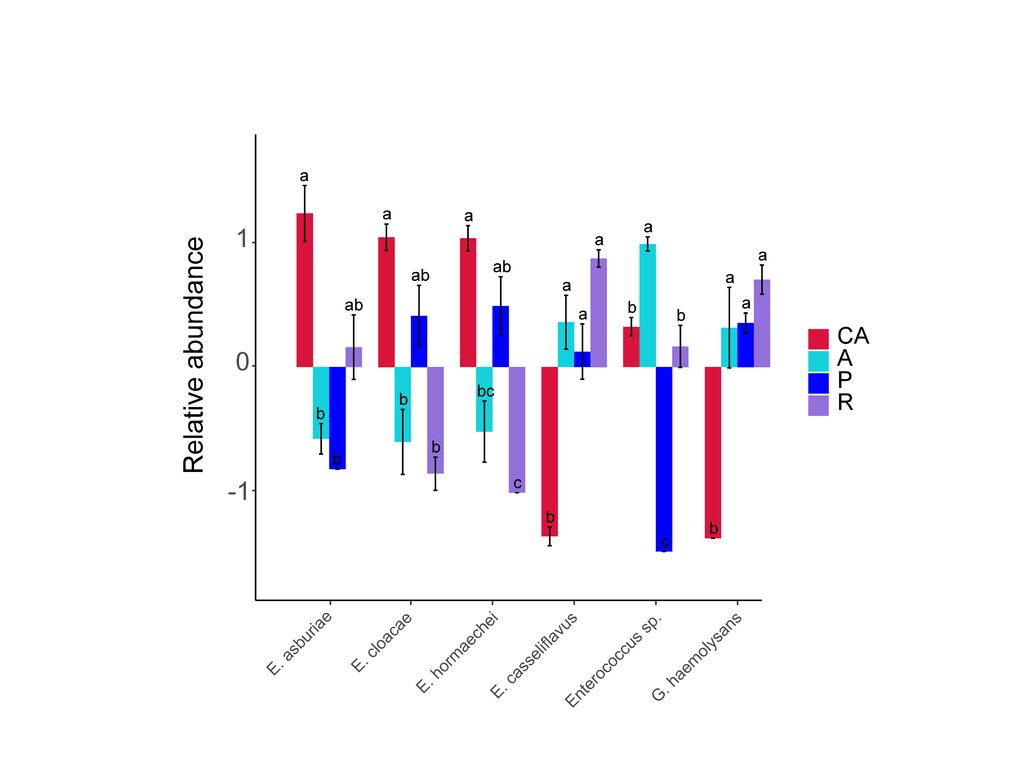
Multiple comparison histogram of intestinal microbiome at the species level. Different lowercase letters on columns indicate significant differences (ANOVA and Duncan test, *P* < 0.05) in the mean values.

We selected six different species and compared the differences of intestinal microbiota amongst feeding groups (Fig. 3). The three species of *Enterobacter* (*E.asburiae*, *E.cloacae* and *E.hormaechei*) had the highest relative abundance in the Chinese ash feeding group (CA). The relative abundance of *E.casseliflavus* and *G.haemolysans* in the Chinese ash feeding group (CA) were higher than that in other feeding groups and there were significant differences between Chinese ash feeding group (CA) and other feeding groups, while there was no significant difference amongst the remaining three feeding groups. The relative abundance of *Enterococcus* sp. contained in the plum feeding group (P) was the highest, followed by the apricot feeding group (A). The relative abundance of *Enterococcus* sp. in the Chinese ash feeding group (CA) and the redbud feeding group (R) was the lowest and there was no significant difference between these two groups.

**Figure 4.**
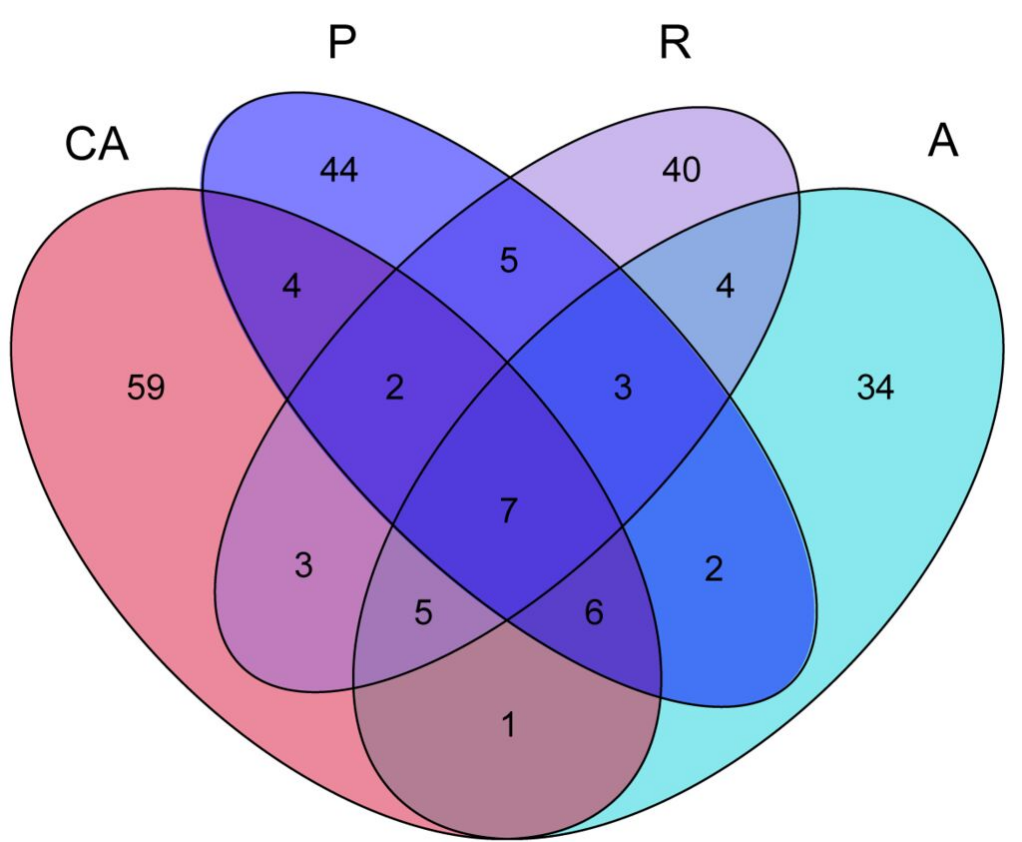
Venn diagram of OTUs from unique species owned by each sample and common species shared by two or more samples.

The OTUs of intestinal microflora of *Hyphantriacunea* larvae in four feeding groups: Chinese ash (CA) feeding group, plum feeding group (P), redbud feeding group (R) and apricot feeding group (A) were compared and 87, 73, 69 and 62 OTUs were annotated, respectively (Fig. 4). Amongst them, the number of OTUs annotated in the Chinese ash feeding group (CA) was the highest and the number of OTUs annotated in the apricot feeding group (A) was the lowest. Only seven OTUs were shared by four groups and the number of OTUs shared between each two groups was small, indicating that there were differences in intestinal microbial communities amongst different feeding groups of *Hyphantriacunea*.

**Figure 5.**
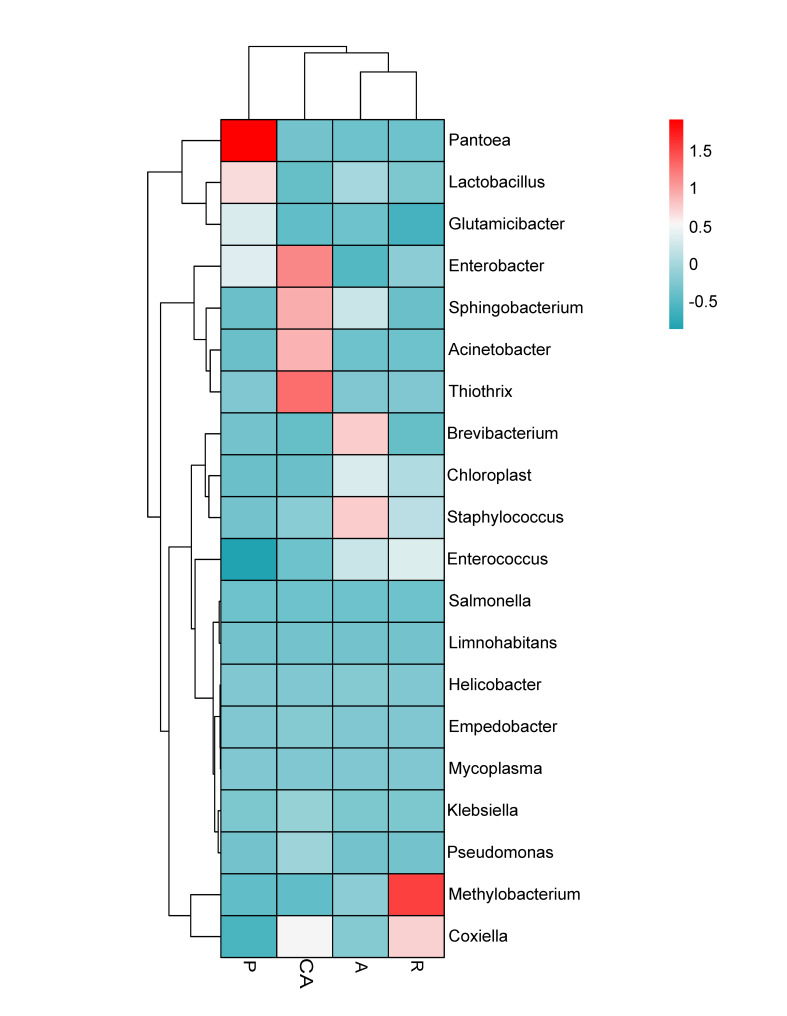
Cluster heat map of the 20 most abundant genera in the bacterial community. The columns represent the samples and the rows represent the bacterial OTUs assigned to the genus level. Dendrograms of hierarchical cluster analysis grouping genera and samples are shown on the left and at the top, respectively.

In order to reveal the dynamic changes of gut microbes, we choose the 20 most abundant genera to draw a cluster heat map. Based on the similarity of species abundance, the cluster heat map can reflect the data information in the two-dimensional matrix through colour change and similarity degree (Fig. 5). Vertical clustering refers to sample information and horizontal clustering refers to species information. The relative abundance of *Brevibacterium* and *Staphylococcus* were the highest in the apricot feeding group (A). In the plum feeding group (P), the highest relative abundance was found in *Pantoea*, followed by *Lactobacillus*. The relative abundances of *Methylobacterium* and *Coxiella* in the redbud feeding group (R) were the highest. *Enterobacter*, *Thiothrix*, *Acinetobacter* and *Sphingobacterium* had the highest relative abundances in Chinese ash feeding group (CA).

**Figure 6.**
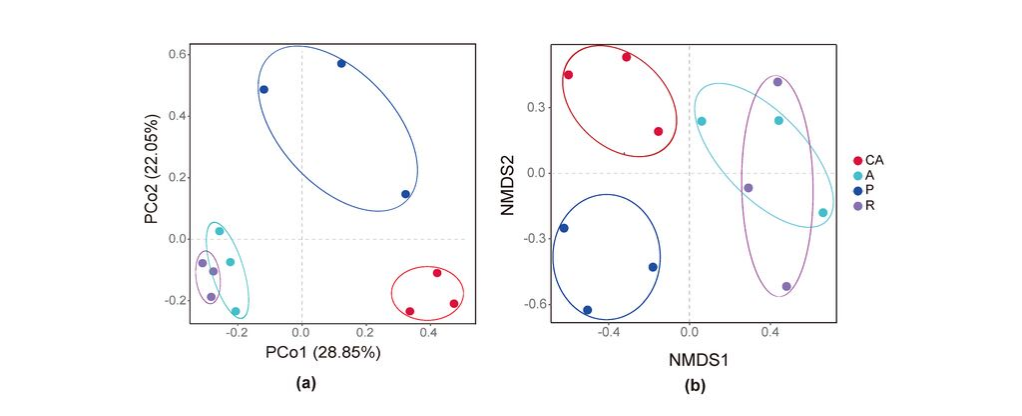
PCoA and NMDS of the gut microbiota of *H.cunea*. (**a**) Principal co-ordinates analysis (PCoA) with Bray–Curtis dissimilarity of the bacterial community between different host diets (**b**) Non-metric multidimensional scaling (NMDS) diagrams of 12 samples, based on the Bray–Curtis matrix. CA, Chinese ash-feeding; A, apricot-feeding; P, plum-feeding; R, redbud-feeding.

In this PCoA scatter plot, the horizontal and vertical coordinates represent the two characteristic values that have the greatest influence on the difference between samples, with the influence degree of 28.85% and 22.05%, respectively (Fig. 6a). PERMANOVA analysis showed that there was no significant difference between the redbud feeding group (R) and the apricot feeding group (A) (PERMANOVA: *F* = 1.453, *P* = 0.4), but there were significant differences between the other feeding groups (PERMANOVA: *F* = 2.928, *P* = 0.001). NMDS analysis based on the Bray−Curtis distance also confirmed this result (Fig. 6b PERMANOVA: *F* = 2.928, *P* = 0.001 metaMDS: stress = 0.127).

**Figure 7.**
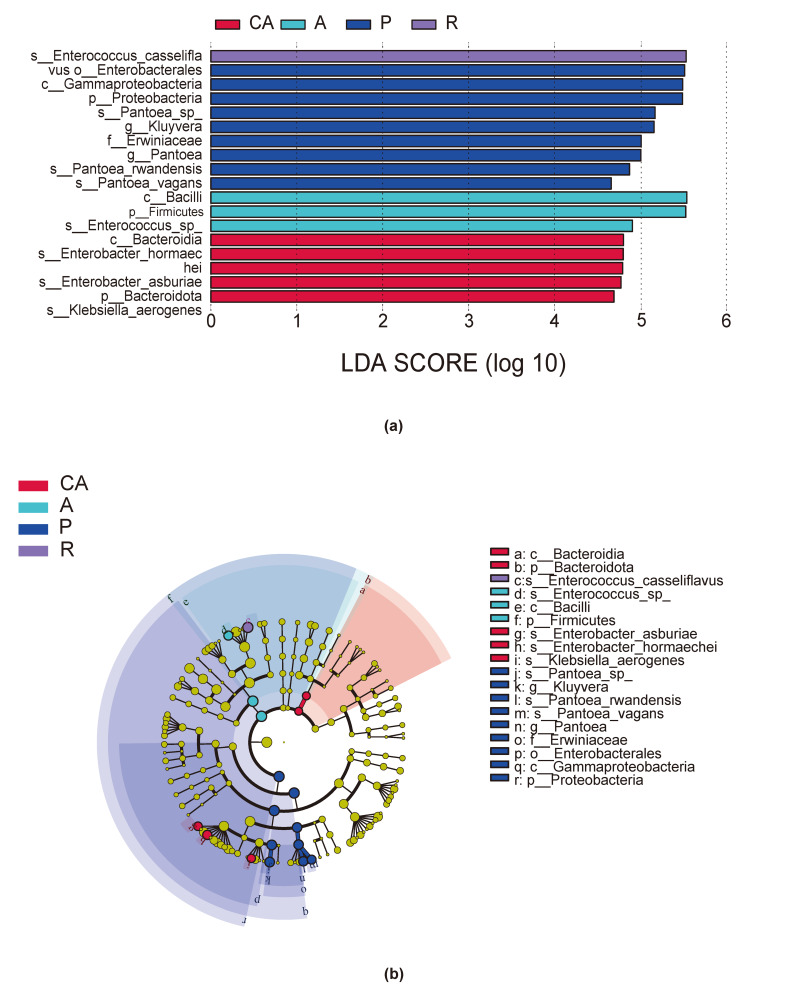
Abundance of different gut microbial taxa observed in the four groups using linear discriminant analysis effect size (LEfSe analysis). (**a**) Bacterial taxa with linear discriminant analysis (LDA) score greater than four in the gut microbiota of *Hyphantriacunea* feeding on different host plants; (**b**) Cladogram of bacterial biomarkers, from the phylum (innermost ring) to species (outermost ring) level, with an LDA score > 4. Differential bacterial taxa are marked by lowercase letters. Each small circle at different taxonomic levels represents a taxon at that level and the diameter of the circle is proportional to the relative abundance. Different colours represent different groups and nodes with different colours represent the communities that play an important role in the group represented by the colour.

To find biomarkers with statistical differences between groups, we used linear discriminant analysis (LDA) effect size (LEfSe) to screen out different levels of taxa (kingdom, phylum, class, order, family, genus and species) between groups, based on standard LDA values greater than four (Fig. 7a). At the same time, we drew the cladogram from phylum to species to fully understand the distribution of these different taxa at different taxonomic levels (Fig. 7b). In the plum feeding group (P), the gut microbiota of *H.cunea* had the most different taxa (LDA > 4), and there were nine taxa, amongst which Enterobacterales was the most highly discriminating taxon. There were significant differences in intestinal microbiota of the Chinese ash feeding group (CA) amongst the five taxa, which belonged to Bacteroidetes and Proteobacteria, respectively. The three taxa in apricot feeding group (A) belonged to Actinobacteria and Firmicutes. Finally, the *Enterococcus* in the intestinal microbiota of redbud feeding group (R) belonged to Firmicutes. Taken together, these analyses confirm the effect of host diet on intestinal community structure, similar to what has been observed in other Lepidopteran herbivores.

**Figure 8.**
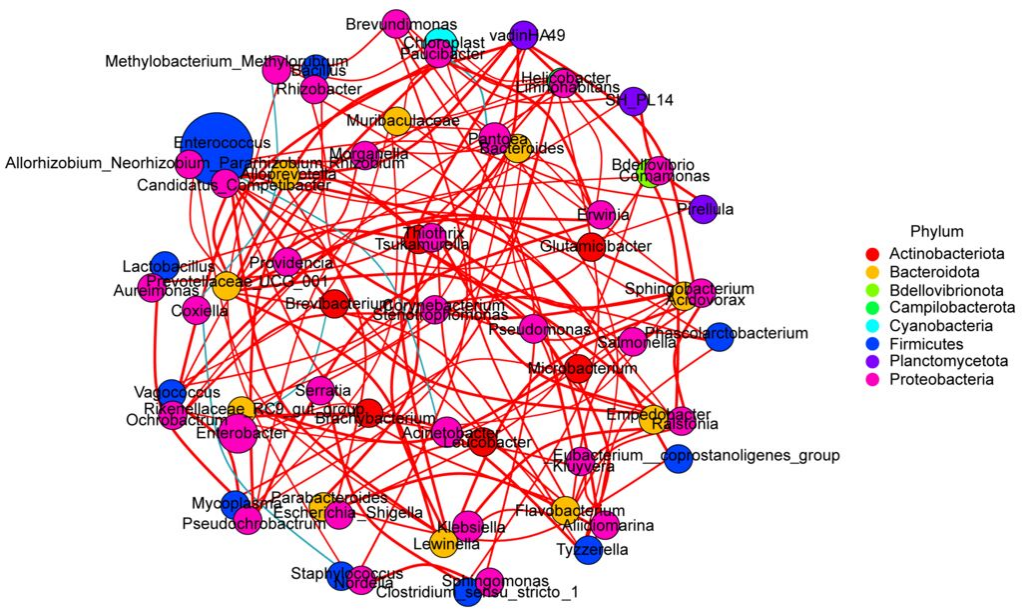
Network analysis of interaction amongst 65 intestinal bacteria genera, based on correlation analysis (Spearman correlation coefficient ρ > 0.5). The node represented unique genera and the size of each node is proportional to the relative abundance. A red edge indicates a positive interaction between two individual nodes, while a blue edge indicates a negative interaction.

To determine the potential relationships amongst bacteria in the gut microbes of *Hyphantriacunea*, we performed a common network analysis, based on sample genus composition (Fig. 8). Firmicutes and Proteobacteria were the dominant phyla and the relative abundance of *Enterococcus* in Firmicutes was the highest.

Most bacteria have positive interactions with each other, that is, the larger the value of one variable is, the larger the value of another variable will be. We speculate that there is a synergistic relationship between bacteria groups with a positive interaction relationship. There were also negative interactions between individual bacteria, that is, the larger the value of one variable was, the smaller the value of another variable was.There were negative interactions between *Coxiella* and *Staphylococcus* (ρ = -0.61), between *Coxiella* and *Methylobacterium* (ρ = -0.51) and between *Enterococcus* and *Acinetobacter* (ρ = -0.56). We speculated that there was an antagonistic relationship between these bacteria.

### Functional prediction of gut microbiota

**Figure 9.**
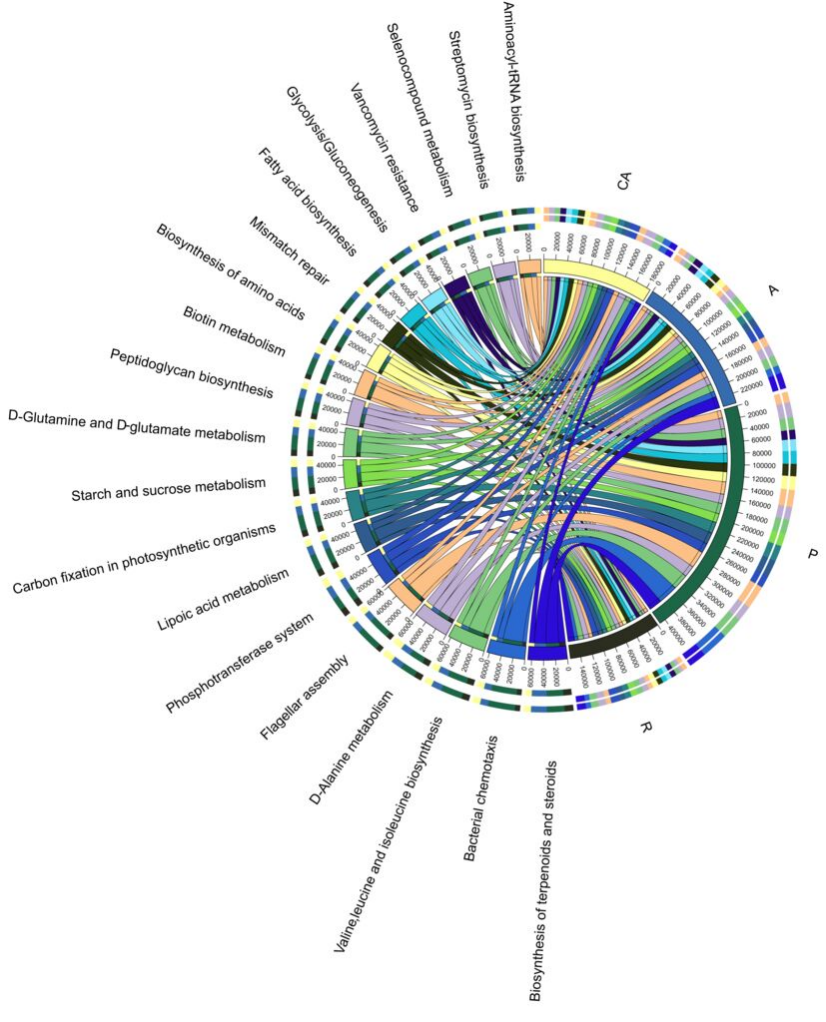
Circos graph of intestinal microbial, based on the KEGG L3 pathway.

In order to better understand the important functions of intestinal microorganisms of *Hyphantriacunea*, PICRUSt2 software was used to predict the composition of functional genes in the samples according to the OTU abundance table and OTU sequence and to draw the Circos graph of intestinal microbial, based on the KEGG L3 pathway (Fig. 9). The results showed that most of the predicted functions were related to metabolic and cellular processes, including biosynthesis, amino acid metabolism, protein transport, mismatch repair etc., representing the most active functions in the intestinal tract.

As the largest functional prediction category, the proportion of terpenoid and steroid biosynthesis was the lowest (1.42%) in the Chinese ash feeding group (CA), but higher in the apricot feeding group (A) (2.80%) and the redbud feeding group (R) (2.65%). The function prediction bar of the apricot feeding group (A) and plum feeding group (P) was relatively higher and the proportion of each function was basically the same in each group. The highest proportion of bacterial chemotaxis (2.52%) and flagella assembly (2.18%) was found in the plum feeding group (P), the highest proportion of peptidoglycan biosynthesis was found in the apricot feeding group (A) (1.74%) and the lowest proportion was found in the plum feeding group (P) (1.2%). The predicted functions with high abundance were terpenoid and steroidal biosynthesis, D-alanine metabolism, phosphotransferase system (PTS), valine, leucine and isoleucine biosynthesis, which were closely related to transport, synthesis, metabolism and cellular processes. These results suggest that the intestinal microbiota of *Hyphantriacunea* plays an important role in accelerating nutrient digestion, transportation and metabolism and signal transduction.

## Discussion

As an invasive species and quarantine pest, *H.cunea* has caused serious impacts on the ecological environment, agricultural and forestry development and social and economic development. For the first time, we used PacBio third generation sequencing technology to sequence the full-length of 16S rRNA gene of the intestinal microorganism of *H.cunea* and detected the intestinal microbe community composition, difference and diversity of *H.cunea* larvae feeding on apricot, redbud, plum and Chinese ash to better understand the relationship between intestinal microbial diversity and feeding habits of *H.cunea*. According to the experimental results, we can preliminarily conclude that the diversity and richness of intestinal microbes of *H.cunea* are affected by feeding habits.

### The composition of insect gut microbes is influenced by a variety of factors, with diet being the most critical

Crotti put forward that operation and development of the microbiome can bring important practical applications for the formulation of management strategies for insect related problems. Specifically, insect microbiome can be manipulated to control agricultural pests, control pathogens transmitted by insects to humans, animals and plants and protect beneficial insects from disease and pressures (Crotti et al. 2012). The composition of intestinal microorganisms is affected by many factors, such as host species, dietary characteristics, health status and living environment. Pyrosequencing of the high-variable region V1-V3 of 16S rRNA gene in the intestinal microflora of *Blattellagermanica* showed that the difference in protein content of food and age of insects affected the structure of intestinal microflora (Pérez-Cobas et al. 2015). By using amplicon sequencing technology, Yuan detected significant changes in intestinal microbial community richness and diversity when *Grapholitamolesta* was transferred from an artificial diet to different host plants (Yuan et al. 2021). By analysing the intestinal flora data of healthy and sick *Phasmotaenialanyuhensis*, it was found that the diversity of intestinal flora and the structure of bacterial community composition changed significantly under different physiological states (Li et al. 2020). In addition, developmental stage also has an important influence on intestinal microbes (Gao et al. 2019, González-Serrano et al. 2020). It has been proved that different dietary habits lead to differences in intestinal microbes (Colman et al. 2012). The intestinal microflora of *Plutellaxylostella* was significantly changed after host transfer (Yang et al. 2020). The intestinal microorganisms of four Lepidopteran fruit pests feeding on different hosts were significantly different (Liu et al. 2020). After eating different prey, the intestinal microbiota of *Badumnalonginqua* changed differently, indicating that the changes of intestinal microbiota are driven by diet and different feeding habits can shape different microbiota (Kennedy et al. 2020).

### The gut microbes of H.cunea play important roles in nutrient acquisition, metabolism and pesticide degradation

At the phylum level, Firmicutes and Proteobacteria were the dominant phyla (Fig. 2a). The dominant phylum in the apricot feeding group (A) and redbud feeding group (R) was Firmicutes, while Proteobacteria was the dominant phylum in the plum feeding group (P) and Chinese ash feeding group (CA). These results are consistent with previous studies on the intestinal microbial diversity of *H.cunea* (Chen et al. 2020) and with the intestinal dominant flora of many other Lepidoptera insects (Mohammed et al. 2018, Bapatla et al. 2018). Members of Proteobacteria, such as *Klebsiella*, have been shown to degrade cellulose (Suen et al. 2018). Firmicutes helps insects digest and obtain nutrients from cellulose, hemicellulose and other polysaccharides (Brown et al. 2012). *Clostridiumsticklandii*, which belongs to Firmicutes, plays an important role in the degradation of amino acids (Yang et al. 2020). The abundance of both *Acetobacter* of Proteobacteria and *Lactobacillus* of Firmicutes increased when *Apismellifera* were fed a diet rich in sucrose and these bacteria can metabolise sugars into monosaccharides and then into acetate (Fonknechten et al. 2010). Proteobacteria could be functionally crucial for insects to adapt to specific host plants (Taylor et al. 2019). These studies suggest that Proteobacteria and Firmicutes may play important roles in host degradation of exogenous substances, metabolism, nutrient digestion and absorption etc.

At the genus level, *Enterococcus* is *the* dominant genus (Fig. 2b). The high relative abundance of *Enterococcus* was considered to protect the host against pathogens and non-commensal microbes from outside and increase the tolerance of a toxic diet by establishing a colonisation resistance effect in mid-gut (Silverman and Paquette 2008, Vilanova et al. 2016). *Enterococcus* has been shown to be abundant in Lepidopteran insects (Liu et al. 2020, Wang et al. 2020) and these results suggest that *Enterococcus* may have some conserved functions in Lepidopteran insects. *Pseudomonas* was annotated the most in the Chinese ash feeding group (CA) and *Pseudomonas* was proved to be able to degrade insecticides and other foreign biological substances (Indiragandhi et al. 2007), suggesting that the larvae in the Chinese ash feeding group (CA) had strong resistance to insecticides. *Acinetobacter* has lignin-degrading activity, while *Staphylococcus* and *Sphingobacterium* have cellulose and/or aromatic degrading ability. Both *Pantoea* and *Klebsiella* belong to the Proteobacteria family *Enterobacteriaceae*, which occur widely in the guts of Lepidoptera and other herbivores and are potentially beneficial, non-pathogenic microbes (Chen et al. 2016). *Pseudomonas* is known to harbour several species able to degrade alkaloids and natural latex or rubber (Vilanova et al. 2016).

### The intestinal microbial composition of H.cunea feeding on different hosts was different and there were complex interactions amongst the different bacteria

PCoA analysis and linear discriminant analysis (LDA) showed that the intestinal microbial composition of *H.cunea* larvae, fed on different hosts, was different (Figs 6, 7). We found that there was significant differences between the Chinese ash feeding group (CA) and other groups, which may be related to the fact that Chinese ash and other plants belong to different families. There are differences in plant morphology, life form, propagation mode, internal structure and genes amongst plants of different families, which lead to differences in nutrients contained in leaves. Interestingly, although plum and apricot belong to Rosaceae, there were significant differences in gut microbes between the two feeding groups. One possibility is that the nutrients in plum leaves are very different from those in apricot leaves. Studies have shown that the oriental fruit moth lays more eggs when using plums as hosts than when using apples and peaches as hosts (Myers et al. 2006). In the study of Yuan, the larva of the oriental fruit moth feeding on plums had the maximum limited growth rate (λ) (Yuan et al. 2021). Wang investigated the feeding selection of *H.cunea* larvae for common garden plants in Suqian area and found that *H.cunea* had a strong selection for plums, indicating that plums are a kind of suitable host (Wang et al. 2020). Host selection by larvae of *H.cunea* may be affected by leaf thickness, leaf wax content, leaf proline content, soluble sugar and soluble protein in leaves (Wang et al. 2020). Further studies are needed to reveal the reasons for the differences in gut microbes amongst feeding groups. Meanwhile, the results of common network analysis on intestinal microorganisms of *H.cunea* showed that most of the bacteria had positive interactions, but there were also negative interactions (Fig. 8). It is limited to use the 16S rRNA gene sequence to analyse the diversity of intestinal microbial community. In the future, we can further study the microbial function by analysing the total genes in the intestinal microbiota of insects, namely metagenomic analysis, to understand the functional mechanism of intestinal microbial community and its impact on the host.

### The prediction results of intestinal microbial function in the plum feeding group were significantly different from those in the other feeding groups

PICRUSt2 analysis showed that the intestinal microbial functions of all feeding groups were similar with minor differences, mainly focusing on biosynthesis, metabolism and material transport (Fig. 9). Amongst them, the functional prediction results of the plum feeding group (P) were significantly different from those of other feeding groups, which was consistent with PCoA analysis results (intestinal microbial community composition of plum feeding group (P) was significantly different from that of other groups). At the same time, the development of omics technology and the application of high-throughput sequencing technology also provide a more effective method to study the molecular mechanism of the interaction between insect gut microbe and insect host.

## Conclusion

Our results confirmed that the gut bacterial structure of *H.cunea* can be influenced by the host plant. The dominant phylum was Firmicutes which fed on apricot and redbud and Proteobacteria which fed on Chinese ash and plum. At the genus level, the content of *Enterococcus* in the plum feeding group was much lower than that in other groups and the content of *Enterobacter* in Chinese ash feeding group was much higher than that in other groups. The cluster heat map showed that the relative abundance of bacteria of different genera in each feeding group was different, indicating that diet influenced the gut microbiome of *H.cunea*. However, there were still many limitations in our study. Some studies have shown that intestinal microbial change is a gradual process (Fonknechten et al. 2010, Crotti et al. 2012), but our experiment only observed one instar of the life stage of *H.cunea*. Future experiments should be designed to observe the changes in gut bacterial communities in the *H.cunea* adaptation to different hosts for successive generations and the important functions of gut bacterial communities in host adaptation should be elucidated with the methods of multi-omics. While using the new technology, we should not abandon the traditional methods of bacterial culture. Our study laid a foundation for further exploring the differences and functions of intestinal microorganisms of *H.cunea* and helped to find new targets for the control of *H.cunea*.

## Data Resources

The raw sequence data reported in this paper have been deposited in the Genome Sequence Archive (Chen et al. 2021) in the National Genomics Data Center (CNCB-NGDC Members and Partners 2022), China National Center for Bioinformation / Beijing Institute of Genomics, Chinese Academy of Sciences (GSA: CRA008201) that are publicly accessible at https://ngdc.cncb.ac.cn/gsa.

### Resource 1

Download URL: Browse - BioProject - CNCB-NGDCBrowse - GSA - CNCB-NGDC

Resource identifier: CRA008201

Data format : FASTQ

## Usage Rights

Usage Rights

## Figures and Tables

**Table 1.** Summary statistics of 16S rRNA sequences from 12 samples of Hyphantriacunea*^1^.

Sample ID	Raw Reads	Filtered	Denoised	Non-Chimeric	Effective (%)
R1	24,754	24,442	23,712	23,712	95.79
R2	11,019	10,857	10,602	10,602	96.22
R3	13,574	13,421	12,989	12,989	95.69
A1	15,107	14,929	14,592	14,592	96.59
A2	25,380	25,054	24,585	24,285	95.69
A3	34,567	34,087	33,534	33,457	96.79
P1	39,238	38,640	37,979	30,046	76.57
P2	35,837	35,219	34,443	31,545	88.02
P3	39,724	39,186	38,466	35,601	89.62
CA1	16,477	16,291	16,023	14,725	89.37
CA2	28,634	28,205	27,608	24,946	87.12
CA3	16,955	16,730	16,163	15,369	90.65

## Footnotes

*1 Raw reads represent the number of original reads sequenced by PacBio. Filter, denoise and non-chimeric are three steps of processing raw data. Effective (%) is the percentage of effective reads in raw reads.
